# Theoretical Prediction of Experimental Jump and Pull-In Dynamics in a MEMS Sensor

**DOI:** 10.3390/s140917089

**Published:** 2014-09-15

**Authors:** Laura Ruzziconi, Abdallah H. Ramini, Mohammad I. Younis, Stefano Lenci

**Affiliations:** 1 Department of Civil and Building Engineering and Architecture, Polytechnic University of Marche, via Brecce Bianche, 60131 Ancona, Italy; E-Mail: l.ruzziconi@univpm.it; 2 Faculty of Engineering, Università Degli Studi e-Campus, via Isimbardi 10, 22060 Novedrate (CO), Italy; E-Mail: laura.ruzziconi@uniecampus.it; 3 Physical Sciences and Engineering Division, King Abdullah University of Science and Technology (KAUST), Thuwal 23955-6900, Saudi Arabia; E-Mails: aramini1@binghamton.edu (A.H.R.); mohammad.younis@kaust.edu.sa (M.I.Y.); 4 Department of Mechanical Engineering, State University of New York at Binghamton, Binghamton, NY 13902, USA

**Keywords:** MEMS sensors, nonlinear dynamic behavior, dynamical integrity analysis, experimental validation

## Abstract

The present research study deals with an electrically actuated MEMS device. An experimental investigation is performed, via frequency sweeps in a neighbourhood of the first natural frequency. Resonant behavior is explored, with special attention devoted to jump and pull-in dynamics. A theoretical single degree-of-freedom spring-mass model is derived. Classical numerical simulations are observed to properly predict the main nonlinear features. Nevertheless, some discrepancies arise, which are particularly visible in the resonant branch. They mainly concern the practical range of existence of each attractor and the final outcome after its disappearance. These differences are likely due to disturbances, which are unavoidable in practice, but have not been included in the model. To take disturbances into account, in addition to the classical local investigations, we consider the global dynamics and explore the robustness of the obtained results by performing a dynamical integrity analysis. Our aim is that of developing an applicable confident estimate of the system response. Integrity profiles and integrity charts are built to detect the parameter range where reliability is practically strong and where it becomes weak. Integrity curves exactly follow the experimental data. They inform about the practical range of actuality. We discuss the combined use of integrity charts in the engineering design. Although we refer to a particular case-study, the approach is very general.

## 1. Introduction

Nonlinear static and dynamic phenomena that may arise in micro- and nano-electromechanical systems have recently received significant and increasing attention from the scientific community. Multistability, jump, chaotic motions, snap-through, pull-in and many other complex nonlinear features represent a very attractive opportunity for improving device performances. New sophisticated micro- and nano-systems deliberately operating in the nonlinear regime are emerging in a variety of different applications, ranging from mass sensors, signal processing and energy harvesting, to health monitoring, laser scanners and bioengineering [1,2]. An extensive overview on current ongoing developments in this field is reported in Rhoads *et al.* [3].

Cho *et al.* [4] fabricated a nanomechanical resonator based on a doubly clamped carbon nanotube incorporating intrinsically geometric nonlinearity. They operate the device in a highly nonlinear regime. Extreme broadband resonance is observed. Both tunability over a broad frequency range, which spans up to many times the device natural frequency, and enhanced sensitivity to external perturbations are explored. Mestrom *et al.* [5] experimentally and numerically investigated a clamped-clamped microbeam-based MEMS resonator. Depending on the excitation parameters, both softening and hardening behaviors are detected. An extensive analysis of the nonlinear response is addressed, which enables parameter study and design optimization of microsystems with respect to nonlinear dynamic features. By using deep-reactive ion etching DRIE, Krylov *et al.* [6] fabricated an electrically actuated microstructure consisting of an arch-shaped microbeam. The arched-shape is properly designed in order that the device is able to exhibit bistable configurations and snap-through motion, with large amplitude robust resonant oscillations, while the resonant frequency may be tuned by the dc voltage. Ramini *et al.* [7] designed a resonant switch for earthquake detection and low-g seismic applications. By careful tuning, the resonator can be made to enter the pull-in instability zone upon the detection of the earthquake signal. Such a switching action can be functionalized for alarming purposes or can be used to activate a network of sensors for seismic activity recording. Hornstein and Gottlieb [8] investigated non-contacting atomic force microscopy and draw a stability map describing the escape bifurcation threshold where the tip “jumps to contact” with the sample. Both periodic, quasiperiodic, and non-stationary chaotic-like oscillations are observed. El Aroudi *et al.* [9] analyzed the bifurcation behavior in a piecewise linear vibrator. A rich variety of nonlinear phenomena is detected, and performances are explored in energy harvesting applications.

Static and dynamic features are currently actively applied in nano-biosensors. We refer to Arlett *et al.* [10] for an extensive review on the most current advances in this topic. The smallness of dimensions perfectly fits with biological issues. These systems offer exceptional sensitivity, which may be applied to measure forces governing biological interactions, displacements, mass-changes from cellular to subcellular processes, *etc*. They discuss examples, explain performances and highlight open challenges. Eom *et al.* [11] analyzed recent developments in nanosystems-based sensing and detection. They highlighted the presence of linear and nonlinear phenomena, recalling the basic principles underlying their use in applications and surveying different modeling approaches.

Many other research studies systematically investigate the bifurcation scenarios, and propose different techniques, based on taking advantage of the nonlinear phenomena. Bahrami and Nayfeh explored grazing dynamics of tapping mode atomic force microscopes [12], Rhoads *et al.* nonlinear features in electromagnetically actuated microbeam resonators [13], Tusset *et al.* chaotic dynamics in MEMS comb-drive actuator [14], Welte *et al.* parametric resonance and anti-resonance [15], Kacem *et al.* primary and superharmonic resonances [16], Vyasarayani *et al.* past pull-in behavior [17], Ouakad and Younis dynamic snap-through motion for filtering applications [18], Corigliano *et al.* the problem of design and reliability assessment of the MEMS response, also in presence of a damage process [19], Garcia and Herruzo multifrequency force microscopy [20], *etc*.

**Figure 1.**
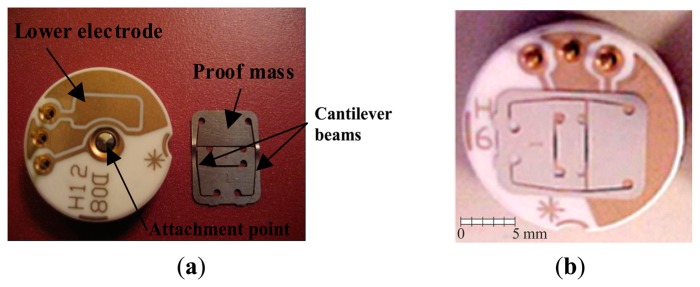
The MEMS capacitive accelerometer, fabricated by Sensata Technologies [33]. (**a**) The device taken-apart; (**b**) The device assembled.

The present research study is encouraged by the growing of practical applications of nonlinearities in MEMS. We consider the MEMS device illustrated in Figure 1, and analyze its response in a neighborhood of the primary resonance, ranging from low up to elevated electrodynamic voltages. This problem is inherently nonlinear and multiphysical. Disturbances, inevitably encountered in practice, add more challenges. They produce small, but non-negligible perturbations, which may significantly affect and alter the system response. Effects of disturbances are observed for instance in Alsaleem *et al.* [21] for a similar MEMS capacitive accelerometer. They observed that the experimental range of existence of each attractor is smaller, and sometimes considerably smaller than the theoretical predictions. Another example is reported in Kozinsky *et al.* [22] for a nanowired-based mechanical resonator. Investigating the nonlinear dynamics of the device, they systematically probe the experimental and theoretical attractor-basins phase portraits, obtaining an excellent matching. Nevertheless, they observe the separatrix defining the boundary of the basins to be smooth in the theoretical simulations, while blurred in the experiment. This is likely due to environmental noise affecting the system and causing uncertainty in the preparation of the experimental initial state close to the separatrix. Predicting the experimental behavior despite the inevitable presence of disturbances is essential for reliable safety estimation of systems load carrying capacity. This issue is deeply theoretically examined by Thompson and coworkers [23,24], and recently by Lenci and Rega [25–27], with applications in a variety of different mechanical systems. Novel theoretical tools are introduced to quantify the integrity of a system against disturbances. Dynamical integrity predictions are recently widely referred in the literature for micro and nanodevices. They are used for interpreting and predicting the experimental behavior [28,29] to model and determine the range of parameters where the device could function effectively. They are referred for getting hints towards engineering design [30,31], in order to understand the nonlinear, multiphysical nature of the MEMS resonator under realistic conditions, experiencing several nonlinear static and dynamic phenomena. Moreover, dynamical integrity simulations play a key role in MEMS research and development for controlling the global dynamics [32].

In the present study, we perform a dynamical integrity analysis of the MEMS response. Our aim is that of developing an applicable confident estimate of the MEMS nonlinear behavior under realistic conditions, which is essential for proper design, performance analysis, and calibration. We provide quantitative information about the loss of structural safety, so that the robustness of a device's MEMS component can be adequately extracted. Special attention is devoted to the practical occurrence of jump and pull-in dynamics, since these nonlinear features are widely applied in practice. For this reason, we systematically explore the bifurcation scenario, with the aim of predicting the parameter range where these features may occur. The outline of the paper is as follows. The MEMS device is introduced (Section 2), investigations are performed (Section 3), dynamical integrity charts are developed (Section 4), and the main conclusions are summarized (Section 5).

## 2. The MEMS Capacitive Accelerometer

In this section we introduce the MEMS device considered in the present study. A single degree-of-freedom spring-mass model is derived to simulate the dynamics in a neighborhood of the first resonance frequency. Experimental data and theoretical results are compared.

### 2.1. Device and Experimental Set-up

The MEMS device investigated in the present paper consists of a proof mass suspended by two cantilever beams. This is a commercial capacitive accelerometer, fabricated by Sensata Technologies [33]. A picture is provided in Figure 1, showing the device, both assembled and taken-apart. The upper electrode is formed by the proof mass, which has a rectangular shape, with length 9 mm, width 5.32 mm, and thickness 150 μm. The lower electrode is placed directly underneath the proof mass on a silicon substrate. It has the same length, but a slightly smaller width, 4.4 mm. The separation gap between the two electrodes is 42 μm. The lower electrode provides both electrostatic and electrodynamic actuation. When electrically excited, the proof mass oscillates in the out-of-plane direction, *i.e.*, out of the plane of the substrate. Although some dimensions are in millimeters, the system has the same main characteristics of a MEMS device, in particular, gap and thickness are in the micro-range and the structure is electrically actuated. There is no dielectric layer. The lower electrode is a ceramic-based material and the upper electrode is metal-based material. These issues help the survivability of this device against stiction and failure from repetition pull-in testing and the heat generated from pulling in. We also add large resistor in series with the short circuit of the device to lower the value of the current passing through the device in the case of pull-in. We have extensively tested the device. According to our experience, the device maintains the same characteristics and the results are repeatable even after large number of tests (meaning the device survives these repeated tests involving pull-in), *i.e.*, differently from other devices, the analyzed capacitive accelerometer has the advantage of being more likely to survive the repetitive failures due to pull-in, which allows a deep experimental investigation.

**Figure 2.**
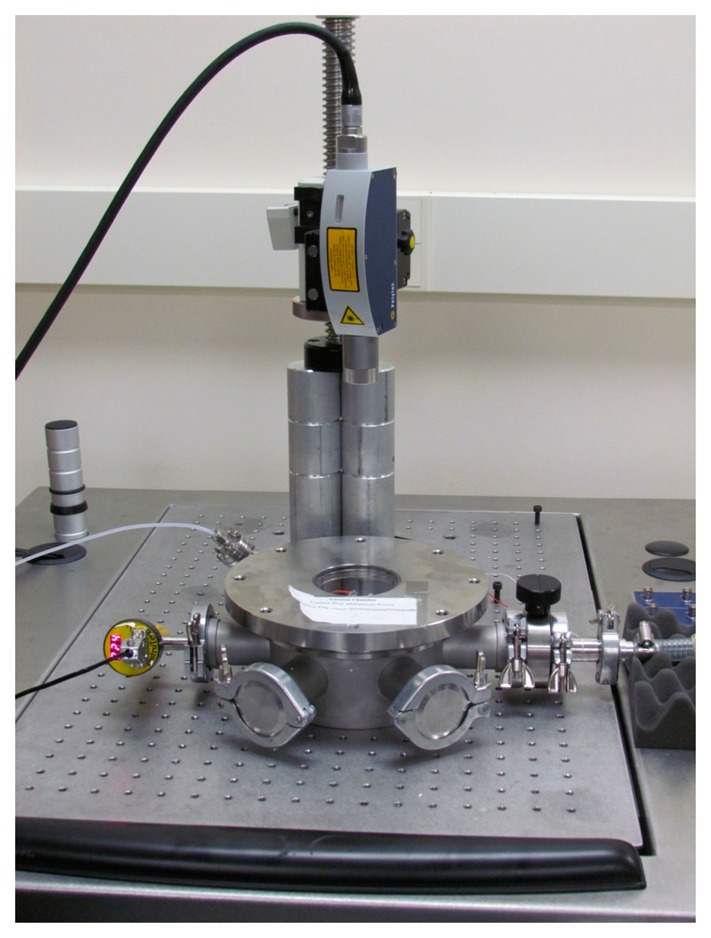
A picture of the experimental set-up used for testing the MEMS capacitive accelerometer showing the laser Doppler vibrometer and the vacuum chamber.

The experimental set-up used for testing the device is represented in Figure 2. It consists of a laser Doppler vibrometer, a Lab View data acquisition system, ac and dc power sources, a vacuum chamber, and a high vacuum pump. The device is inserted inside the vacuum chamber, which is placed underneath the laser Doppler vibrometer, such that it can measure the deflection of the proof mass. The chamber is equipped with a viewport window, located on top and made of quartz glass, and with some ports, located in the lateral sides. The viewport window enables the laser to penetrate without any distortion. The lateral ports, instead, serve to supply pressure gauge and electrical connection. They are used to hook the chamber up to the high vacuum pump and to apply the ac and dc power sources, which are provided via the Lab View data acquisition system. The signal is generated using DAQ card and applied on the outer pins shown in Figure 1. A power amplifier is used to amplify the signal, if needed.

### 2.2. Model Formulation

**Figure 3.**
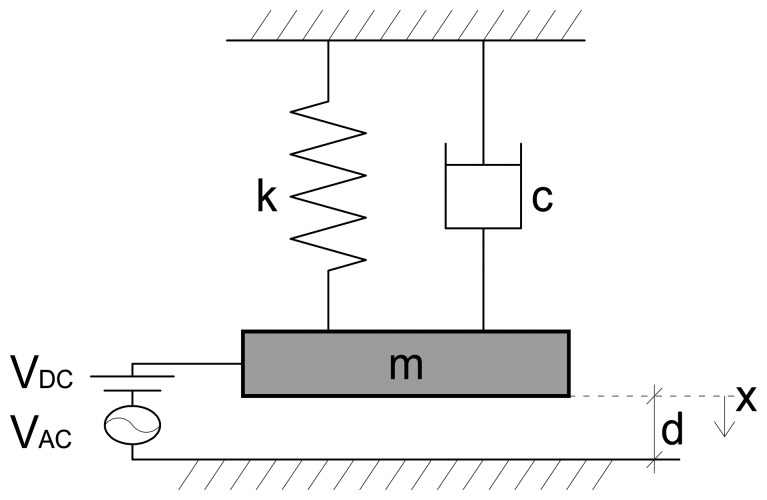
Single d.o.f. mechanical model used to model the capacitive sensor.

The interest of this work is for vibration near the first resonance frequency (primary resonance). To describe the device nonlinear response in this range, a single degree-of-freedom spring-mass model is considered, which is schematically illustrated in Figure 3. The capacitive sensor is modelled as a parallel plate capacitor with two rigid plates, where the upper one is movable. The lumped mass represents the proof mass, and the spring represents the two cantilever beams. The resulting governing equation of motion is:

(1) $$m\ddot{x}+c\dot{x}+kx={ɛ}_{0}{ɛ}_{r}A\frac{{\left[V_{\text{DC}}+V_{\text{AC}}\cos(\Omega t)\right]}^{2}}{2{\left(d-x\right)}^{2}}$$

where *x* is the deflection of the proof mass, *m* is its mass, *c* is the viscous damping coefficient due to the squeeze film effect, *k* is the linear effective stiffness of the cantilever beams, *ε_0_* is the dielectric constant in the free space, *ε_r_* is the relative permittivity of the gap space medium (air, *ε_r_* = 1) with respect to the free space, *A* is the lower electrode area, *d* is the separation gap width including the static effect of the mass weight, *V_DC_* and *V_AC_* are respectively the electrostatic and electrodynamic voltage, Ω is the electrodynamic voltage frequency, *t* is the physical time, the superscript dot denotes the time derivative. Since the size of the proof mass is very big compared to the gap width underneath, it is safe to assume negligible fringing and to suppose straight lines electric field.

We have used the same model for this device in several other previous papers [34,35], in which we compared theoretical results to experiments and found excellent agreement among all. All the assumed assumptions (parallel plate capacitor with a moving plate, presence of squeeze film damping, parameter extractions, *etc.*) have been examined carefully in our previous works, to which we refer for more details. Hence, we have good level of confidence in the model. In the following, we briefly recall the main steps for its formulation, since this is the starting point of the forthcoming analysis.

The unknown parameters of Equation (1) are: *k*, *m*, and *c*. To extract the stiffness coefficient *k*, we resort to the static bifurcation diagram, and match experimental and theoretical predictions. In particular, we bias the microstructure with ramping *V_DC_* inputs and we measure the stable static deflection of the proof mass, up to the static pull-in phenomenon. Static pull-in voltage *V_DC_pull-in_* is observed at about 115.3 *V*. Focusing on the spring-mass model Equation (1) in the static case, from simple computations, it can be easily proven that the stiffness coefficient *k* is:

(2) $$k={ɛ}_{0}{ɛ}_{r}A\frac{{\left[V_{\text{DC}\_\text{pull}-\text{in}}\right]}^{2}}{2d^{3}(4/27)}$$

Based on these considerations, we identify *k* = 215 N·m^−1^.

To determine the effective mass *m* of the proof mass, we focus on the first natural frequency, which experimentally occurs at about 192.5 Hz. Recalling that *m* = *k*/*ω^2^*, the effective mass of the proof mass is estimated, *m* = 0.147 g.

Regarding the damping, various mechanism of energy dissipation may affect the device. Among them, squeeze-film damping dominates all other damping mechanisms in MEMS and may be considered the main source of energy loss [2]. In our device, this is even more pronounced since the proof is very large compared to the gap width, and hence it pumps and sucks considerable air in and out (rubbing against the walls of the plate causing energy loss). Hence it is safe to assume that the only damping is coming from squeeze-film damping. The damping coefficient *c* is computed by means of the Blech model, which analytically solves the linearized Reynolds equation with trivial pressure boundary conditions. We consider only the first term in the series of the Blech model, since higher order terms do not affect the damping value too much because the pressure is very low and the air is not trapped underneath the movable mass. Thus:

(3) $$c=\frac{768\eta_{\text{eff}}PaA^{2}}{\pi^{6}{\left(d-x\right)}^{3}}(\frac{2}{\left[4+\sigma^{2}/\pi^{4}\right]})\;\text{with}\;\sigma =\frac{12A\Omega \eta_{\text{eff}}}{\text{Pa}{\left(d-x\right)}^{2}}$$

where *P_a_* is the ambient pressure, *i.e.*, the pressure value of the device during its operation, which is here 43 mtorr, and *η_eff_* is the effective viscosity coefficient of air, which in this case is *η_eff_* = 4.34 × 10^−8^ Ns/m^2^. The gap space in Equation (3) is varying with the proof mass motion. In the following we assume the gap space between two electrodes to be constant and equal to *d*, *i.e.*, we drop the dependence on *x* in Equation (3). This is sufficiently accurate for our purposes, as proven experimentally in various previous publications on the same device [34,35]. This yields: *c* = 3.45 × 10^−4^ (Ns)m^−1^.

For convenience, we divide Equation (1) by the extrapolated value of *m*. The resulting governing equation of the nonlinear dynamics of the MEMS device becomes:

(4) $$\ddot{x}+2.344\dot{x}+1.459\cdot 10^{6}x=1.2\cdot 10^{-12}\frac{{\left[V_{\text{DC}}+V_{\text{AC}}\cos(\Omega t)\right]}^{2}}{{\left(42\cdot 10^{-6}-x\right)}^{2}}$$

where *x* is expressed in micron. Equation (4) is the single d.o.f. model used in the forthcoming numerical investigations. The model parameters are summarized in Table 1.

As mentioned above, the validity of this model has been examined extensively in previous research [34,35]. Of course, more complicated models may further improve the results, e.g., by introducing other loss mechanisms, by supplying more terms in the Blench model, by adding the effect of fringing fields, by catching more resonances, *etc.* However, simplicity in the model is desirable for engineering-oriented investigations, *i.e.*, for the targets of this study. Also, for the purpose of the dynamical integrity analysis, which is the focus of this work, simple and accurate models are required to carry the intensive computational work involving, as better observed next, the calculations of attractor-basins phase portraits and dynamical integrity measure. Hence, since this single d.o.f. model has proven to be accurate, there is no need to complicate further the model to improve accuracy. For all these reasons, although many improvements may be performed, in the present paper we prefer referring to this simple model, which, without detriment to the accuracy, is able to properly catch all the essential aspects without being cumbersome.

**Figure 4.**
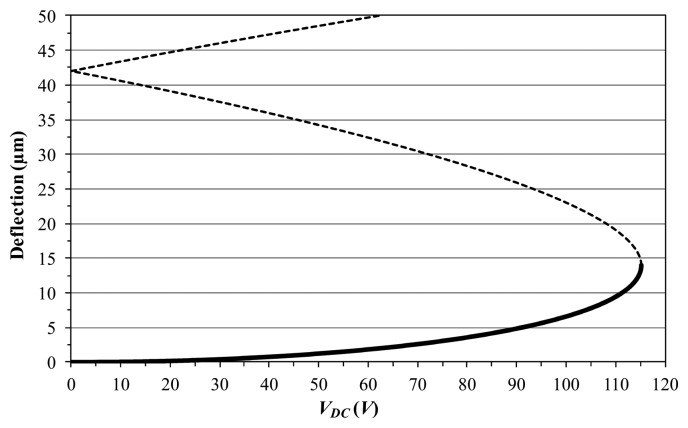
Maximum static deflection *versus* electrostatic voltage *V_DC_*.

In the following, Equation (4) is solved based on direct integration by resorting to the Runge-Kutta method. As preliminary investigation, we report in Figure 4 the maximum static deflection *versus* the electrostatic voltage.

### 2.3. Experimental Data

**Figure 5.**
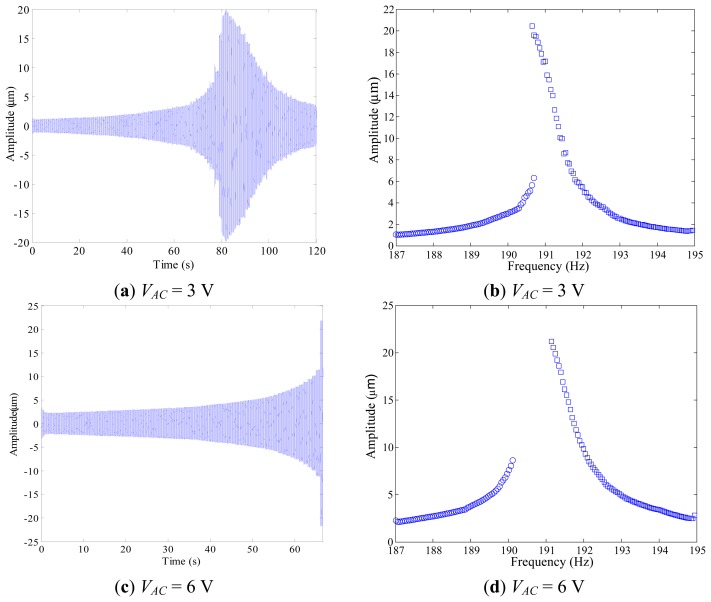
Experimental time history of the forward frequency sweep, and experimental frequency response diagram with forward and backward sweep in blue dots and squares, respectively:

We investigate the nonlinear dynamic features arising in the system response when both the frequency and the electrodynamic voltage are varying, by analyzing the device behavior in a neighbourhood of the first resonance. Several frequency sweeps are performed. Each sweep is acquired by keeping the electrodynamic voltage *V_AC_* as constant, while the frequency is increased (forward sweep) or decreased (backward sweep) slowly, to guarantee the steady-state condition at the end of each step. Two examples are reported in Figure 5. Note that, to compare the sweeps among them, they are attained by adopting the same experimental conditions, in particular: (i) *V_DC_* = 20 V, *i.e.*, the electrostatic voltage is kept constant and is small enough not to produce particularly relevant static deflection (Figure 4); (ii) pressure is 43 mtorr, *i.e.*, the experiment is conducted close to an ultra-high vacuum environment; (iii) frequency step is 0.05 Hz, *i.e.*, we implement the same frequency step in all the sweeps.

We report in Figure 5a the experimental forward frequency sweep at *V_AC_* = 3 V. Step by step, we can observe the typical resonant behavior, with jump from the non-resonant to the resonant oscillations. To better visualize the dynamics without the transient effect, we plot the corresponding frequency response diagram, Figure 5b, which is obtained by removing the transient and recording the maximum amplitude of oscillations occurring at each step. Both the forward and the backward sweep are reported, respectively in blue dots and squares. Following the forward sweep, the non-resonant branch (left hand side of the curve) slowly increases up to about Ω = 190.7 Hz. Then there is a jump to the resonant branch (right hand side of the curve). After that, the oscillation slowly decreases up to achieve the starting values. The backward sweep shows that experimental jump occurs also from the resonant branch to the non-resonant one. In this case, jumps practically take place at about the same frequency value, *i.e.*, using the aforementioned experimental conditions we cannot clearly perceive any substantial range of coexistence of the two branches.

Increasing *V_AC_*, the scenario qualitatively changes. As an example, Figure 5c shows the experimental forward frequency sweep at *V_AC_* = 6 V. Increasing the frequency, the amplitude of oscillations increases, but, differently from the previous case, no jump is finally observed. The device gets in touch with the substrate and directly undergoes dynamic pull-in. Once pulled-in, the laser measurements become inaccurate. Also, because of the impact with the substrate, it seems the laser point shifts from near the tip of the proof mass to other parts of the device. The frequency response diagram in Figure 5d clearly shows that dynamic pull-in occurs in the backward sweep as well. We can clearly distinguish a parameter range where no bounded attractors are observed, which spans from about Ω = 190.1 Hz up to about Ω = 191.2 Hz, *i.e.*, in this interval only dynamic pull-in may be expected in the present experiment. Note that knowing in advance where jump and pull-in dynamics may arise is useful, since several applications take advantage of them and many different research studies propose approaches and techniques, which make use of these nonlinear features to build sensors, switches, filters, *etc.* [2].

## 3. From Local to Global Investigations

In this section, simulations are compared to experiments. It is observed that disturbances unsurprisingly give uncertainties to the operating initial conditions. If the response is not sufficiently robust against disturbances, a small shift in the initial conditions may lead to a completely different outcome. To interpret discrepancies, it is essential analyzing the behavior not only locally, by studying each single attractor, but also globally, by focusing on the attractor-basin scenario.

### 3.1. Theoretical Results vs. Experimental Data: the Inevitable Presence of Disturbances

**Figure 6.**
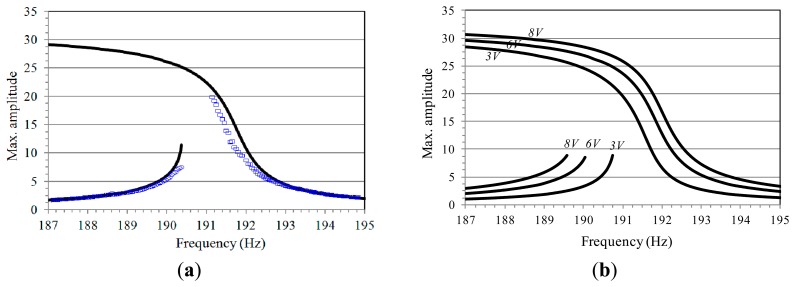
(**a**) Frequency response diagram at *V_AC_* = 5 V. Theoretical simulations are in black solid line. Experimental forward and backward sweep are in blue dots and squares, respectively (for convenience, the last part of the theoretical resonant branch is denoted in dashed line); (**b**) Theoretical frequency response curves at *V_AC_* = 3 V, *V_AC_* = 6 V, *V_AC_* = 8 V.

Referring to model Equation (4), we focus on the device frequency response and analyze similarities and discrepancies of the theoretical predictions with respect to the experimental data. To this purpose, we simulate the theoretical frequency response, where the maximum amplitude of oscillation is illustrated, and we compare them with the experimental frequency sweeps. An example is reported in Figure 6, at *V_AC_* = 5 V. The theoretical maximum amplitude of the oscillations is slightly scaled, since experimental dynamic measurements were not done exactly on the tip of the mass where the amplitude is maximum. Both the resonant branch, and the non-resonant one, and the separation width between them are properly described, *i.e.*, the considered theoretical model is able to simulate with satisfactory accuracy all the main features of the experimental response.

Nevertheless, the extent of each branch is longer in the simulations. The theoretical non-resonant attractor disappears by saddle-node bifurcation (SN) at about Ω = 190.4 Hz, whereas the resonant one extends for a wide range, which exceeds the analyzed interval. The experimental disappearance, instead, occurs where the attractor is theoretically expected to exist and a clear gap (in frequency) between the experimental branches is observed, *i.e.*, simulations are not able to forewarn with sufficient accuracy the experimental vanishing, especially in the resonant case, but predict a broad range of coexistence, which is not observed in practice.

This mismatching is likely related to disturbances in the experiments, which are inevitably encountered. For instance, the theoretical frequency response diagram is constructed from the governing equation Equation (4) and relies on its numerical time integration until the steady state motion is reached with initial conditions equal to the solution for the previous frequency value. To catch the whole theoretical branch, numerical simulations require both a very small frequency step, which can be approximated to an infinitesimal one, and a sufficiently long time interval in order to settle on the attractor and investigate the dynamics at steady-state. A small frequency step and a large time step are especially necessary in the vicinity of the bifurcation points, because, as better explained next, in this region the basin of attraction is very narrow. In the present simulations we use a frequency step of 0.005 Hz and we analyze dynamics after 50 (or more) forcing cycles each step. On the contrary, the experimental frequency step is small but cannot be approximated as infinitesimal, as assumed to be in the theoretical predictions. Similarly, the removed experimental transient before assuming steady-state is smaller than in the simulations. This is because we cannot ignore the limitations of the experimental set-up, e.g., restrictions in the amount of data that can be saved for each sweep, which directly affects our choice on the magnitude of the steps in frequency and time.

Discontinuities in the sweeps are just one source of disturbances, but many others may occur. In addition, when building a theoretical model, approximations are inevitably introduced. They cannot be avoided as well. Here we report some relevant examples. Both effective mass and stiffness are not measured, but only extrapolated. When performing experiments, the pressure is not perfectly constant as assumed in the theoretical formulation, but slightly varies. Hence, even if we were very careful in the experiments in reducing disturbances as much as possible, the system is inevitably exposed to them. They are not accounted in the model, likely giving rise to the aforementioned discrepancies. Similarly occurs at higher voltages.

**Figure 7.**
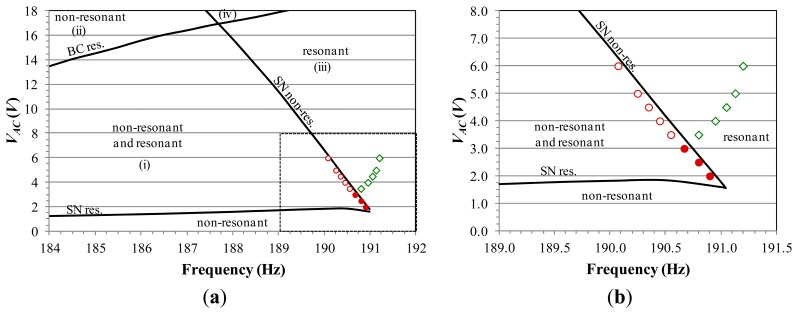
(**a**) Frequency-dynamic voltage behavior chart. Theoretical disappearance is in black line. Experimental disappearance is in red dots and green diamonds, respectively for the non-resonant and the resonant sweep. Solid and empty symbols respectively denote jump and pull-in dynamics; (**b**) A zoom of the analyzed parameter range.

For completeness, the theoretical frequency response is simulated at *V_AC_* = 3 V, *V_AC_* = 6 V, *V_AC_* = 8 V, Figure 6b. To have a comprehensive and detailed description of the overall scenario when both frequency and dynamic voltage are varied, we build the frequency-dynamic voltage behavior chart in Figure 7, where theoretical bounds of existence of each attractor are overlapped to the experimental ones.

To construct the experimental part of the chart, many frequency sweeps are acquired. Collecting information from these sweeps, we report the (Ω, *V_AC_*) values where each attractor experimentally disappears. The non-resonant branch is marked in red dots, whereas the resonant one in green diamonds. Since we wish to describe the final behavior obtained at the disappearance of each attractor, we use solid and empty symbols, respectively to denote jump and pull-in dynamics. Coexistence between branches is actually inexistent, *i.e.* all these cases present the disappearance of an attractor overlapping to the appearance of the other one. Up to *V_AC_* = 3 V, jump dynamics arise both from the non-resonant oscillations and from the resonant ones. At *V_AC_* ≥ 3.5 V, instead, each branch directly leads to dynamic pull-in. Each attractor disappears when the other attractor does not exist. The resulting outcome is an increasing *V*-shaped region, where no bounded motions are visible. This is initially small, and then enlarges up to span a wide frequency range.

Regarding to the theoretical part of the chart, this is obtained by following the same procedure used for the experimental data, except that information is extracted from the theoretical frequency response diagrams. We can observe the same main characteristics arose in the experimentation, but shifted (considerably shifted in some cases). In the neighbourhood of the primary resonance at low electrodynamic voltage, there is the degenerate cusp bifurcation, which occurs at about (Ω, *V_AC_*) equal to (191; 1.58). At this point, the non-resonant and the resonant attractor separate, and start developing their curves of appearance and/or disappearance (SN non-resonant and SN resonant, respectively). Accordingly, the non-resonant attractor exists all along the parameter region starting from the unforced dynamics up to its SN bifurcation curve. The resonant attractor, instead, starts from its SN curve at about *V_AC_* = 1.5 V, develops for a wide range, and finally disappears by boundary crisis (BC resonant). We can distinguish: (i) a Δ-shaped region with both the non-resonant and the resonant attractor, which is bounded by the SN bifurcation of the non-resonant branch, and by the SN and the BC of the resonant one; (ii) a region with only the non-resonant attractor, which is located in the left hand side of the chart; (iii) a region with only the resonant one, which is located in the right hand side of the chart; (iv) a *V*-shaped region with vertex at about (Ω, *V_AC_*) equal to (187.7, 17), where no bounded motions may occur and dynamic pull-in (*i.e.*, escape) is inevitable.

The chart highlights the same aforementioned discrepancies detected in the frequency response diagrams. In particular, the experimental range of existence of each branch is smaller than theoretically predicted; the major difference emerges in the resonant one, where the shape of the experimental band of disappearance relevantly differs from the theoretical one; according to the theoretical simulations, both the non-resonant and the resonant attractors exist in the Δ-shaped region, whereas in practice no range of coexistence is observed, but the disappearance of a branch coincides with the appearance of the other one.

To analyze the final outcome after the disappearance of the attractors, theoretical information is extracted by simulating the frequency response at many different increasing *V_AC_* voltages, and by recording if, when an attractor disappears, the theoretical response shows a jump to the other branch or leads to the escape. Relevant dissimilarities arise also in this case: theoretically, safe jump occurs up to *V_AC_* = 6.3 V in the non-resonant branch and up to *V_AC_* = 2 V in the resonant one; experimentally, instead, dynamic pull-in is observed in both branches at *V_AC_* ≥ 3.5 V.

Therefore, despite the good matching using Equation 4, some discrepancies are observed. This is because disturbances inevitably occur in the experiment. They produce small, but relevant perturbations. However, they are not taken into account in the theoretical investigations previously reported. In the forthcoming simulations our aim is that of developing a theoretical study which is able to take into account also the unavoidable presence of disturbances. This is essential to reliably predict the final behavior of the device. To this purpose, we need to examine the system not only via local investigations, as presented in the previous analysis, but also from a global perspective, by developing a basin of attraction analysis and by introducing dynamical integrity concepts to assess the robustness of the basins. This analysis is in addition (not in substitution) to the classical one.

### 3.2. Global Investigations

Before developing dynamical integrity investigations, it is worth specifying that we refer to the same theoretical model. To clarify this point, let us consider an example, where we suppose an experimental frequency sweep of the MEMS device. Initially, we assume it to be performed with a certain pressure and a certain step in the time and in the frequency. We will obtain a certain experimental frequency response curve, with a certain length of the resonant branch. Then, we suppose to repeat the same experiment, with the same device, the same pressure, the same electric excitation, but with a smaller step both in frequency and in time. Experimentally, we will obtain a longer resonant branch. Nevertheless, we have not modified the device parameters, *i.e.*, the constants in the equations have not changed:

– we have the same device, *i.e.*, the same spring and mass coefficients in the equation
– we have the same pressure, *i.e.*, the same damping coefficient

We have just varied disturbances. Since they are not related to coefficients but to uncertainties in the initial conditions, we do not need to improve the model. Keeping the same model, instead, we need to analyze it more in depth by studying the system from a global viewpoint.

**Figure 8.**
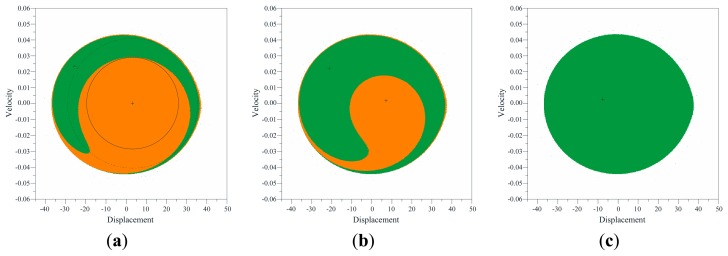
Attractor-basins phase portraits at *V_AC_* = 3 V and (**a**) Ω = 189.5 Hz; (**b**) Ω = 190.5 Hz; (**c**) Ω= 192.0 Hz. Example of circles used in the definition of LIM are in solid and dashed lines, respectively referring to the analysis in Section 4.1 and in Section 4.2.

**Figure 9.**
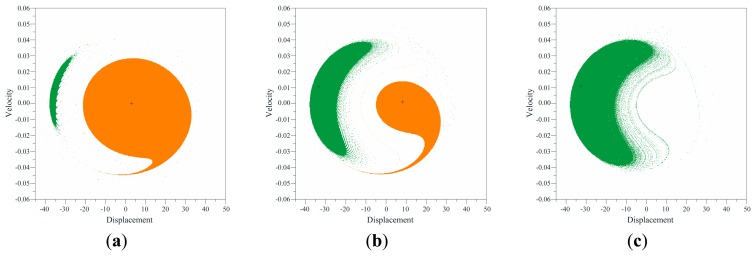
Attractor-basins phase portraits at *V_AC_* = 8 V and (**a**) Ω = 187.0 Hz; (**b**) Ω = 190.0 Hz; (**c**) Ω = 190.5 Hz.

For this reason, many attractors-basins phase portraits are computed, where displacement *versus* velocity is reported. Some significant examples are shown in Figures 8 and 9. They are obtained by using the software package Dynamics [36] whose results are confirmed also by a lot of investigations via self-developed codes. By comparing the obtained results among them, we can achieve a detailed description of the phase space metamorphoses when varying the electrodynamic excitation. At low values of *V_AC_*, only the non-resonant attractor exists. Increasing the voltage, instead, the resonant branch appears and competes in robustness with the non-resonant one. An example is observed in Figure 8, which summarizes the global behavior at *V_AC_* = 3 V. The basins of the non-resonant and of the resonant branch are orange and green, respectively; escape is white; attractors are denoted with a cross. Far from resonance, at Ω = 189.5 Hz (Figure 8a), the basin of the resonant attractor is not particularly wide. It is located close to the escape area, surrounding the other basin. The basin of the non-resonant branch, instead, is very large, and develops around the attractor. A wide compact area is essential to tolerate disturbances. Conversely, a small one is vulnerable. Approaching the resonance, at Ω = 190.5 Hz (Figure 8b), the basin of the resonant branch enlarges, at the expense of the other basin, which gradually shrinks in size, up to disappearance. At Ω = 192.0 Hz (Figure 8c), only resonant motion exists, with a wide compact basin. Slightly above the natural frequency, the resonant attractor is eccentric with respect to its basin. Further increasing the frequency, instead, eccentricity sensibly reduces and actually vanishes. Note that, all the analyzed attractor-basins phase portraits show the two basins close to each other, *i.e.*, at this stage, they form a large compact area, which is not affected by the escape.

Increasing the voltage, the scenario qualitatively changes. We analyze for instance the global dynamics at *V_AC_* = 7 V, Figure 9. Both the non-resonant and the resonant oscillations keep existing, but the escape enters the potential well with fractal tongues and completely surrounds and separates the basins. At Ω = 187.0 Hz (Figure 9a), the basin of the resonant branch is very reduced, and the safe area surrounding its attractor is residual. The non-resonant branch, instead, has a large basin. The separation between the basins prevents any safe jump, e.g. when the resonant attractor disappears, its basin is replaced by the escape, leading to dynamic pull-in, and not by the other basin, even if this one still exists and is robust. Approaching the natural frequency, at Ω = 190.0 Hz (Figure 9b) and Ω = 190.5 Hz (Figure 9c), the basin of the non-resonant branch shrinks and finally disappears. The basin of the resonant one, instead, progressively enlarges, even if the attractor remains eccentric as far as the excitation frequency is close to the resonance.

## 4. Robustness and Vulnerability of the Response

In this section, we develop a dynamical integrity analysis to address the discrepancies highlighted in Section 3, and we examine both the practical disappearance of each attractor (Section 4.1) and the practical final outcome (Section 4.2).

### 4.1. Practical Disappearance of Attractors

To explore the practical disappearance of each attractor, we analyze each single branch separately. We choose the dynamical integrity tools of safe basin and integrity measure that ensure compliance with the experimental frequency sweep. In particular, since we are interested in the disappearance of each single attractor, we consider as safe basin the basin of attraction of each single attractor, *i.e.*, safe dynamics are represented by having at steady-state the branch under consideration, whereas unsafe dynamics are represented by having at steady-state all the other motions, both the bounded (in this case, the other branch) and the unbounded (escape) ones.

As regards to the integrity measures, many different definitions are proposed in the literature. We refer to Lenci and Rega [25,26] for a complete overview on this topic. In the present paper, we consider the Local Integrity Measure (LIM) introduced by Soliman and Thompson [24]. LIM is the normalized minimum distance from the attractor to the boundary of the safe basin, *i.e.*, the radius of the largest circle entirely belonging to the safe basin and centered at the attractor. Examples of circles used in the definition of LIM are reported in Figure 8 in solid line. Note that the LIM is an appropriate measure for our case-study. This is because it directly investigates the scenario close to the steady-state dynamics (in fact, the circle used in the computation is centered at the attractor), *i.e.*, LIM directly focuses on the compact core of the safe basin surrounding the attractor, which we are interested in. Conversely, LIM does not include in the computation as safe areas the fractal parts, which are dangerous from a practical point of view. We normalize each radius in the definition of LIM with respect to the analogous radius drawn for the non-resonant attractor at the unforced dynamics (however, other different normalizing conditions may be considered; results will be just scaled).

**Figure 10.**
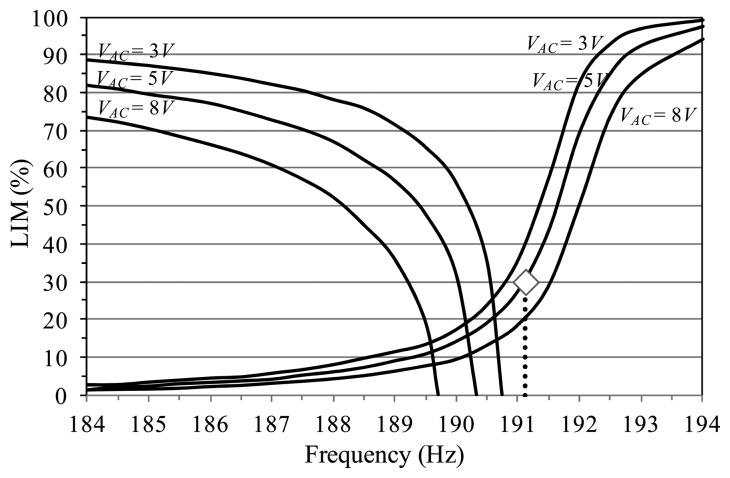
LIM integrity profiles to analyze the practical disappearance of the non-resonant (at left) and the resonant (at right) branch. Experimental disappearance at *V_AC_* = 5 V is in green diamond.

We explore the changes of LIM dynamical integrity while the excitation parameters are varied by building several LIM integrity profiles. Operatively, we consider a certain fixed *V_AC_* voltage, perform attractor-basins phase portraits at different increasing values of frequency (ranging from Ω = 184 Hz up to Ω = 194 Hz, with step ΔΩ = 0.5 Hz or smaller), compute the LIM for each attractor (*i.e.*, the ratio between the radius in the considered case and the radius in the normalizing condition), and draw the integrity profile, where we report LIM *versus* frequency. Some of them are illustrated in Figure 10 in solid lines. For completeness, Table 2 shows some examples of LIM dynamical integrity at different voltage and frequency excitations, which have been used to build the profiles.

We start by examining the resonant branch, since this is the attractor where the major discrepancies arise. We consider for instance *V_AC_* = 5 V, but the same sequence of events does not qualitatively vary when changing the voltage. We can clearly distinguish three different parameter regions. Above the natural frequency (right hand side of the profile), LIM dynamical integrity is really elevated. There is a nearly constant plateau, where LIM reaches and overcomes LIM = 90%, *i.e.*, the resonant branch is very robust against disturbances. This is due to the large basin of attraction of the attractor. Experimentally, the attractor is clearly visible in practice all along this Ω-range, despite the presence of substantial disturbances. Approaching the resonance (middle part of the profile), a sudden fall occurs. LIM quickly drops and switches from LIM = 70% at Ω = 192 Hz up to LIM = 10% at Ω = 189.5 Hz, *i.e.*, LIM abruptly becomes practically residual. From an attractor-basins point of view, this is related to fractality, which increasingly erodes the basin of attraction of the attractor, drastically decreasing the safe area. This is the Ω-range where the attractor experimentally disappears, *i.e.*, is not adequately robust to tolerate experimental disturbances. Further decreasing the frequency (left hand side of the profile), the basin of attraction is almost completely fractal and its magnitude actually trivial. LIM additionally reduces and gradually leads to the complete disappearance of the attractor. This is in agreement with the experiments, since in this range we are not able to catch the resonant branch. Comparing the integrity profiles with the frequency response diagrams, we can perceive that only small oscillations are paralleled with a large structural safety. When the amplitude increases, robustness drops, vulnerability critically amplifies until the attractor practically vanishes. For instance, in the resonant branch, when the oscillations enlarge their amplitude (at about Ω < 191.1 Hz, Figure 6), LIM drastically falls (LIM < 30%, Figure 10). The final range is only theoretical, and cannot be seen in practice, since its LIM is irrelevant in this interval.

Increasing and/or decreasing the voltage, the main features illustrated above do not qualitatively change. Similarly occurs in the non-resonant attractor, with the exception that, when LIM starts dropping, it straight leads to the disappearance of the branch, without the extended range of tiny LIM.

**Figure 11.**
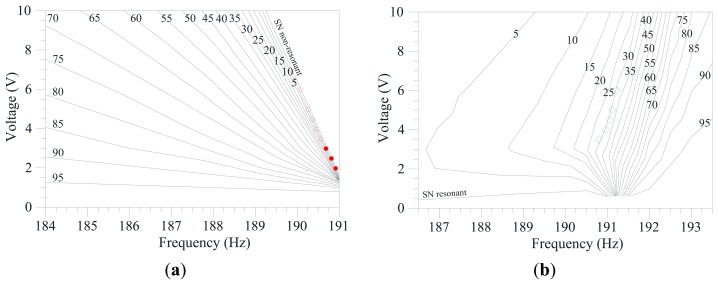
LIM integrity chart to analyze the practical disappearance for the (**a**) non-resonant and the (**b**) resonant branch. Experimental disappearance is in red dots and green diamonds.

To have a comprehensive outline of the overall response when both frequency and dynamic voltage are varied, we assemble information coming from all the performed integrity profiles and build the integrity chart in Figure 11. This is obtained by self-developed codes implemented in Mathematica. The experimental data are overlapped to the theoretical iso-LIM lines. For both the non-resonant and the resonant case, the chart shows many integrity curves, each one corresponding to a certain constant percentage of LIM. They span from complete reliability up to total vulnerability. Interesting matching is observed in the non-resonant branch (Figure 11a), but even more astonishing in the resonant one (Figure 11b). Here, we can clearly distinguish the three different ranges detected in the integrity profiles. There is the range with elevated LIM, which is growing and/or shrinking gradually. It corresponds in the chart to a wide interval with a slow development of integrity curves (right hand side of the chart). Decreasing the frequency, this is followed by a rapid occurrence of many integrity curves, which corresponds to the sudden fall in the LIM integrity profiles. Finally (left hand side of the chart) integrity curves develop gradually again and progressively lead to the theoretical curves of appearance and/or disappearance (*i.e.*, LIM = 0%). The experimental bands of disappearance follow exactly the LIM integrity curve. They share the same shape, which is qualitatively different from the curves of appearance and/or disappearance. Safe conditions are ensured at LIM > 30%. Below this percentage, the attractor becomes vulnerable and practically vanishes. Similarly arises in the non-resonant branch, where safety is ensured up to about LIM > 20%.

Thus, the LIM integrity chart can be used to detect a safe threshold, below which the device becomes vulnerable in practice. Obviously, this threshold differs for each mechanical system under consideration, but it appears to be fixed for a given one: here 30% for the resonant branch and 20% for the non-resonant one, whatever the applied voltage. This is remarkable enough to be noted as it validates the use of LIM, and, more in general, of the dynamical integrity analysis, as an indicator of the amount of uncertainty in the operating initial conditions and in the model.

The practical range of existence is a subset of the theoretical one. Discrepancy may be considerable, especially in the resonant branch. Integrity charts provide a valuable theoretical tool to predict the experimental behavior. They inform about the practical range of actuality, which is fundamental in applications. Moreover, the chart is a valid reference for the engineering design, since collects all the integrity curves, *i.e.*, may be used to reliably design the device when different expected disturbances may occur and to set adequately safety targets.

### 4.2. Practical Jump and Pull-in Dynamics

**Figure 12.**
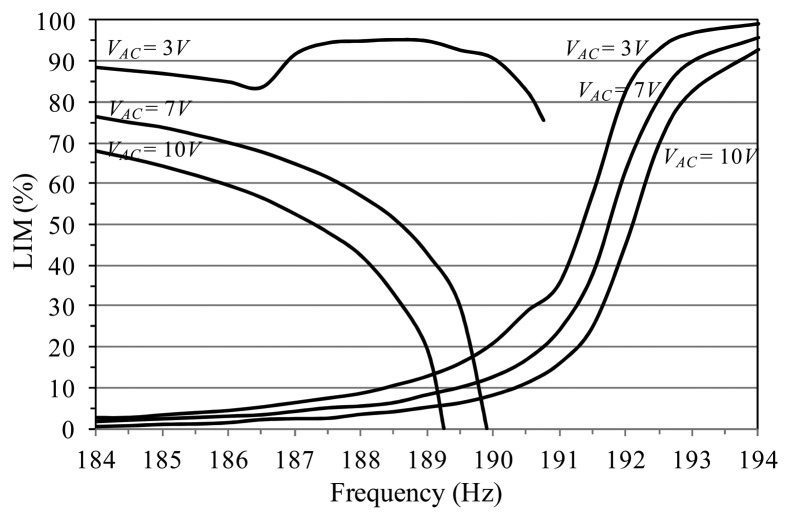
LIM integrity profiles to analyze the practical jump and pull-in dynamics.

In this section we explore jump and/or pull-in dynamics arising in practice after the disappearance of a certain attractor. Similarly to the previous analysis, we investigate each single branch separately and consider LIM dynamical integrity. Differently from the previous analysis, in this case we consider as safe basin all the basins of attraction of bounded attractors. This is because safe dynamics are represented by having at steady-state a safe bounded motion, whereas unsafe dynamics are represented by having at steady-state dynamic pull-in. Examples of circles are reported in Figure 8 in dashed line. Some integrity profiles are illustrated in Figure 12.

We consider the non-resonant branch, since this is the attractor where we can observe the major differences with respect to the previous analysis. At small *V_AC_* (e.g., at *V_AC_* = 3.0 V), LIM is really elevated. Contribution to its magnitude derives both from the basin of attraction of the analyzed attractor and from the basin of the resonant branch, which surrounds it. When the attractor disappears in practice, its basin is replaced by the other basin, producing jump from non-resonant to resonant oscillations, as experimentally observed.

Increasing the voltage, the surrounding area reduces and finally escape enters and separates the two basins (e.g., at *V_AC_* = 10 V). In this case, the safe basin is represented only by the basin of attraction of each single attractor. Consequently, integrity profiles exactly coincide with the previous ones in Section 4.1. When the attractor practically disappears, there are no other resources of safety, but dynamic pull-in is the only possible outcome. A similar outline occurs for the resonant case, except that its basin is typically closer to the escape, and, for this reason, the safe area surrounding the attractor is smaller and mainly due to its own basin of attraction.

**Figure 13.**
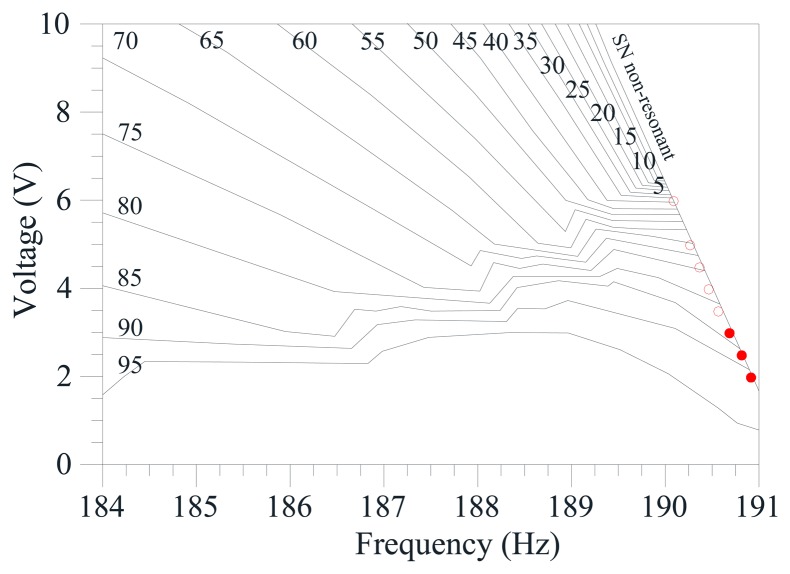
LIM integrity chart to analyze the practical jump and/or pull-in dynamics for the non-resonant branch. Experimental jump and pull-in are in solid and empty symbols, respectively.

Building the LIM integrity chart, we can observe three different regions, Figure 13. At about *V_AC_* < 3.0 V, LIM dynamical integrity is really elevated (LIM > 85%) up to the practical disappearance of the attractor. This guarantees the safe jump between branches. Increasing *V_AC_*, at about 3.0 V < *V_AC_* < 6.0 V, LIM drastically drops. In the attractor-basins phase portraits, this corresponds to the penetration of the escape inside the potential well. Consequently, the safe area may be not robust enough to guarantee a safe jump, as observed in the experiments. Finally, at about *V_AC_* > 6 V, the analysis overlaps to the previous one in Figure 11a, *i.e.*, when the attractor disappears, only dynamic pull-in is expected. The experimentation confirms these predictions.

Summarizing, the combined use of the integrity charts offers a complete understanding of the dynamics under realistic conditions. The analysis in Section 4.1 alerts where an attractor is expected to disappear in practice. The analysis in Section 4.2, instead, forewarns if the practical disappearance will finally end up with a safe jump from an attractor to the other one, or with dynamic pull-in.

## 5. Summary and Conclusions

We have considered a MEMS capacitive accelerometer, which consists of a proof mass resonating due to ac and dc power sources. By using a laser Doppler vibrometer, we have experimentally tested the device in a neighbourhood of the first resonance. Several frequency sweeps have been acquired, and both jump and pull-in dynamics have been detected. Based on the experimental data, a single d.o.f. spring-mass model has been developed.

An extensive analysis has been performed, which is mainly focused on the range where simulations have an experimental counterpart. We have observed that classical investigations are able to catch all the main nonlinear features of the response. Nevertheless, some relevant discrepancies have been highlighted. They concern both the length of each branch, and the final outcome after its disappearance. To have a comprehensive description, we have developed the behavior chart, where both theoretical and experimental bounds of existence of the non-resonant and the resonant branch have been reported, when both electrodynamic voltage and frequency are varied. We have noticed that the experimental bands of disappearance of each attractor do not coincide with the theoretical ones. They are shifted from them, and occur in the region where each attractor is expected to exist. The shift is minimal in the non-resonant case, but considerable in the resonant one. Similarly, dynamic pull-in has been experimentally attained at different excitations amplitudes than anticipated. All these discrepancies are likely due to the presence of disturbances, which are unavoidable in practice, as discontinuous steps when performing the sweeps and fluctuation in the pressure. Taking disturbances into account is essential for safety estimation of the device response.

Here comes the importance of overcoming local investigations and exploring dynamics from a global perspective, by introducing dynamical integrity concepts. Their aim is that of providing a simple but accurate and efficient interpretation of the presence of disturbances, which have not been considered in the traditional simulations. To this purpose, we have continued referring to the same simple and easy to handle theoretical model, but, in addition to the classical local study, we have developed a global analysis where attractor-basins phase portraits have been deeply examined. We have observed that a wide compact area of the safe basin is essential to tolerate disturbances and reliably operate the device. Conversely, a non-compact region is sensitive to them, because a small shift in the initial conditions may lead to a different outcome. To have a quantitative estimate of the MEMS structural safety, we have developed a dynamical integrity analysis. Both the definition of the safe basin and the integrity measure have been selected in order to establish a parallelism with the experimental conditions of the sweeping process. Integrity profiles have been systematically developed, and findings have been summarized in the integrity charts. When LIM drops, the attractor becomes practically vulnerable and may not tolerate disturbances. Accordingly, the final motion may become different from the classical theoretical predictions, as experimentally observed. Extensive dynamical integrity simulations have been performed. In addition to the analysis of the disappearance of attractors, both jump and pull-in dynamics have been explored, in view of the increasing applications of these nonlinear phenomena in practice. The apparent qualitatively different shape of the experimental and theoretical bands of disappearance has been justified. Experimental data exactly follow the integrity curves, and not the classical theoretical predictions. Also, we can detect a safe threshold, below which the device becomes vulnerable in practice. This validates the theoretical approach.

We have emphasized that integrity charts offer a valuable guideline for the engineering design. They inform about the practical range of actuality, which is a very important characteristic in applications. After making assumptions for the magnitude of the expected disturbances, the combined use of the charts may serve to properly predict the experimental response. Also they may be used to establish safety targets, in order to safely operate the device according to the desired outcome. Ranges of applicability of the obtained results as well as limitations are specified. Finally, it is worth noting that, even if the paper has referred to a particular system, the approach is very general.

## Figures and Tables

**Table 1.** Model parameters.

**m (g)**	**k (Nm^−1^)**	**c (Ns)m^−1^**	**d (m)**	**Pa (mtorr)**
0.147	215	3.45 × 10^−4^	42 × 10^−6^	43

**Table 2.** LIM dynamical integrity to analyze the disappearance of the resonant attractor.

	184.0	184.5	185.0	185.5	186.0	186.5	187.0	187.5	188.0	188.5	189.0	189.5	190.0	190.5	191.0	191.5	192.0	192.5	193.0
2	2.81	3.24	3.40	3.45	4.32	4.48	5.18	5.39	6.15	6.69	7.34	7.77	7.88	11.65	42.18	67.53	89.75	96.01	98.49
3	2.54	2.59	3.24	3.78	4.32	4.64	5.61	6.58	7.88	9.60	11.33	13.27	17.26	23.84	35.71	57.61	82.74	92.88	96.87
4	2.27	2.59	3.13	2.91	3.67	4.21	5.07	5.83	7.23	8.31	10.57	12.41	15.86	21.58	30.96	49.84	76.05	89.64	94.28
5	1.40	2.16	2.37	3.02	3.34	3.78	4.21	5.29	6.15	7.34	9.06	10.90	14.24	19.20	27.62	43.69	69.47	85.01	92.23
6	1.62	1.94	2.27	2.59	2.91	3.24	4.10	4.96	5.39	6.26	8.20	10.03	12.62	16.83	24.16	37.97	63.43	80.80	89.97
